# Oriented Collagen Scaffolds for Tissue Engineering

**DOI:** 10.3390/ma5030501

**Published:** 2012-03-16

**Authors:** Yoshihiro Isobe, Toru Kosaka, Go Kuwahara, Hiroshi Mikami, Taro Saku, Shohta Kodama

**Affiliations:** 1 Atree Inc., 16-12-1 Hiroo Shibuya-ku, Tokyo, 150-0012, Japan; E-Mails: isobe@a-tree.co.jp (Y.I.); t-kosaka@a-tree.co.jp (T.K.); saku@a-tree.co.jp (T.S.); 2 Department of Regenerative Medicine & Transplantation, Faculty of Medicine, Fukuoka University, 7-45-1, Nanakuma, Jonan-ku, Fukuoka City, Fukuoka Pref, 814-0133, Japan; E-Mails: kuwahara@fukuoka-u.ac.jp (G.K.); mikkan0805@mac.com (H.M.)

**Keywords:** oriented collagen, three-dimensional, scaffold, biomaterial, tissue engineering

## Abstract

Oriented collagen scaffolds were developed in the form of sheet, mesh and tube by arraying flow-oriented collagen string gels and dehydrating the arrayed gels. The developed collagen scaffolds can be any practical size with any direction of orientation for tissue engineering applications. The birefringence of the collagen scaffolds was quantitatively analyzed by parallel Nicols method. Since native collagen in the human body has orientations such as bone, cartilage, tendon and cornea, and the orientation has a special role for the function of human organs, the developed various types of three-dimensional oriented collagen scaffolds are expected to be useful biomaterials for tissue engineering and regenerative medicines.

## 1. Introduction

The extracellular matrix (ECM) found in the human body plays a central role in controlling cell behavior such as migration and proliferation. The most prevalent protein in this complex structure is collagen. Because collagen possesses a major advantage in being biodegradable, biocompatible, lack of immunogenicity issues, easily available and highly versatile, the use of collagen-based biomaterials in the field of tissue engineering applications has been intensively growing [1].

The application fields of collagen-based biomaterials cover wide range of fundamental studies both *in vivo* and *in vitro* such as bone and cartilage reconstructions [2,3,4], vascular diseases [5,6,7], wound healing [8,9,10], cornea regenerations [11,12,13], urogenital system [14,15], peripheral nerve regenerations [6,16] and drug delivery systems [17].

Because native ECMs extracted from tissue and organs have an oriented morphology, attention has also been given to the connection between substrate orientation and cell morphology, adhesion, and proliferation. Many applications in regenerative medicine require scaffolds characterized by specific orientational order. Bone [18,19], muscle [20], and nervous tissue [21,22] are examples of systems where the orientation of cells and extracellular matrix material directly influences the ultimate physical properties of the resulting tissue, or is essential for proper performance of that tissue.

A review of the literature shows that anisotropic films and gels of collagen fibrils have been produced using several methods such as dip-pen nanolithography [23], reverse dialysis [24], high strength magnetic field application [16,25,26,27], and electrospinning of a fibrous mat onto a spinning disk [28]. These techniques often require complex equipment and may not be the most convenient processes suitable for commercial production. Moreover, the techniques are neither flexible to produce any size nor any direction of orientation of collagen scaffolds for three-dimensional environments in tissue engineering.

In the present study, oriented collagen scaffolds were developed in the three-dimensional form of string, sheet, mesh and tube by arraying flow-oriented collagen string gels and dehydrating the arrayed gels. The size of the scaffolds and the direction of orientation are easily controllable. The oriented collagen strings were prepared using hydrodynamics to influence the assembly of collagen fibers. Two types of collagen solution, rat tail type-I and porcine skin type-I, were subjected to shear and extensional flow as they are drawn onto a substrate under phosphate buffered saline (PBS) buffer. The orientation of the collagen scaffolds were analyzed and confirmed by optical birefringence measurement.

## 2. Materials and Methods

### 2.1. Materials

Rat tail collagen, type-I (BD Biosciences, USA) and pepsin solubilized porcine skin collagen type-I (Nippi, Japan) were used at a relatively high concentration of 10 mg/mL in 0.02 N Acetic Acid (pH~3.5). The buffer used to induce fibrillogenesis of collagen molecules was 10× PBS (Gibco, Invitrogen Corporation, USA). The buffer has a molarity of 0.1 and pH of 7.4. The PBS was heated to 37 °C before use.

### 2.2. Methods

Oriented collagen gels in the form of string were prepared based on the previous study [6,29]. Then, other types of form such as sheet, mesh and tube were designed by arraying the string type gels and dehydrating the arrayed gels.

The gels in the form of string were produced by using hydrodynamic flow to influence the formation of collagen fibers by orienting the molecules of collagen in solution prior to their entry into a neutral pH PBS buffer. The collagen solution is deposited under the PBS, inducing fibrillogenesis of the collagen molecules following the exit from the needle orifice. During the deposition process, the fluid is subjected to two flow profiles, the shear flow of the fluid ejected from the syringe, and the extensional flow of the fluid in contact with the substrate being pulled in the opposite direction.

**Figure 1:**
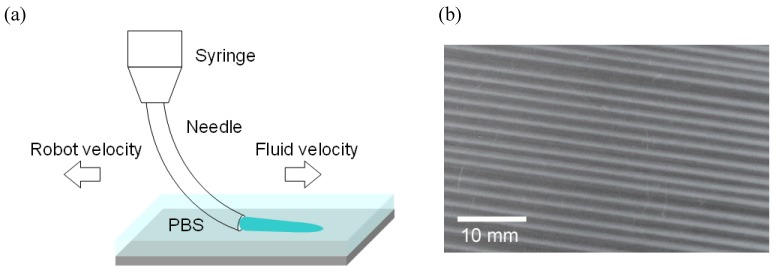
(**a**) Schematic of robotic deposition under PBS buffer, (**b**) and the deposited oriented collagen gel strings.

Collagen strings were created using a deposition system that includes a three axis robotic arm (SM300–3A, Musashi Engineering, Japan) and a syringe. The design of the apparatus allowed the deposition of the collagen solutions onto a variety of substrates by programming the robotic arm to follow the path of the substrate surface. The arm of the robot supports a disposable syringe needle connected to an external air compressor and a pressure regulator, which dispenses the collagen solutions. The syringe needle is a flexible polypropylene 22 gauge needle (inner diameter: 0.38 mm) so that the exiting fluid is ejected parallel to the target surface as shown in Figure 1. The opposing directions of fluid and robotic movement create an extensional flow component in the fluid exiting the syringe.

**Figure 2:**
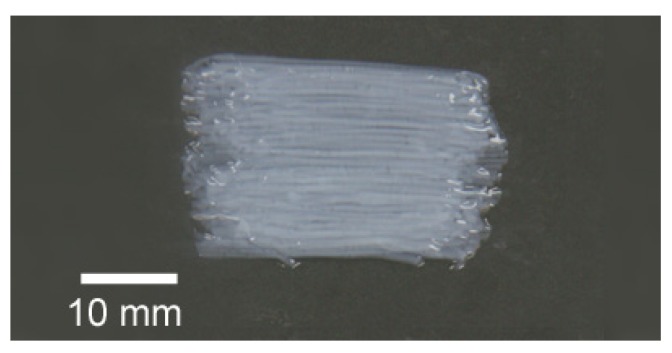
Arrayed oriented collagen gel strings to prepare an oriented collagen sheet.

The deposition speed of the robotic arm was set at 550 mm/s and the air pressure to the collagen solution was 0.1 MPa. The length of the straight string gels was 200 mm, although the robotic arm itself can travel up to 300 mm. Adjusting the needle gauge, the deposition speed and the air pressure can vary the diameter of the string gels and the degree of orientation of collagen scaffolds as well. The collagen gel strings were kept under PBS buffer for at least 5 minutes after the deposition and they were removed from the PBS buffer. Then the strings were rinsed with distilled water for demineralization and arrayed on a polypropylene film to form a sheet (Figure 2). Dehydration of the sheet was conducted at ambient conditions. Dehydrated oriented collagen sheets can be easily removed from the surface of the film by alternating bending of the film. The three-dimensional size and the direction of orientation of collagen scaffolds are controllable when arraying the string gels.

## 3. Results and Discussion

### 3.1. Three-Dimensional Oriented Collagen Scaffolds

**Figure 3:**
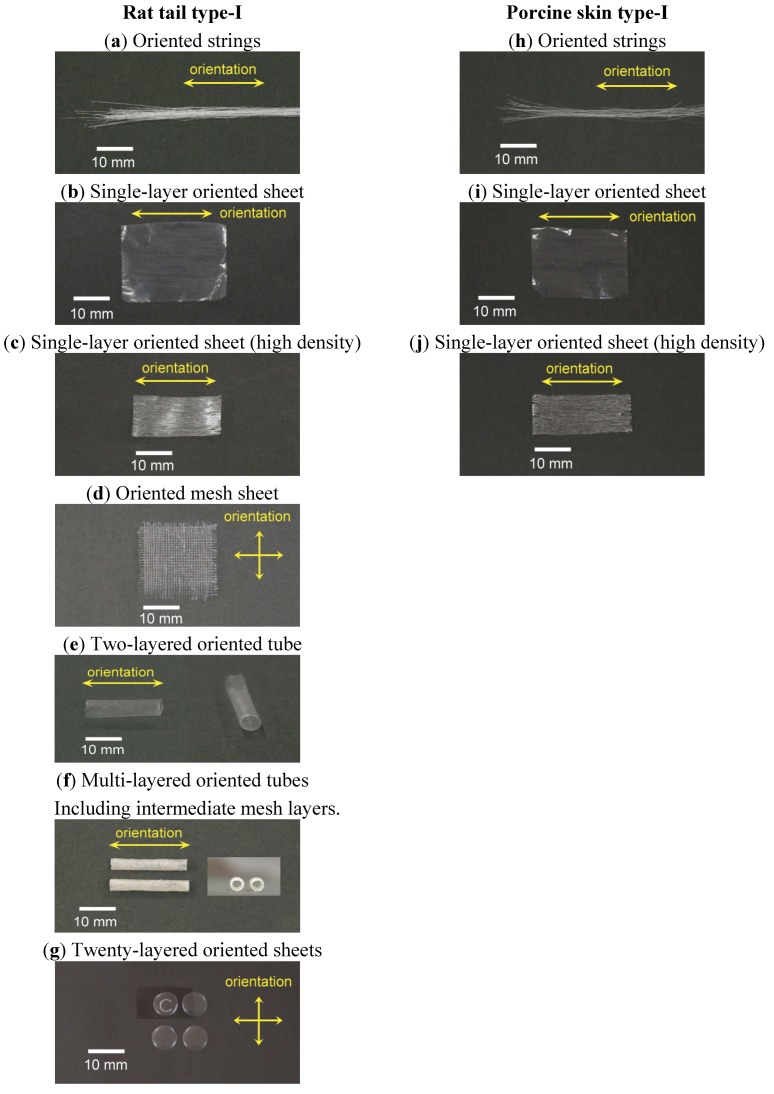
Various types of dehydrated oriented collagen scaffolds prepared with rat tail type-I and porcine skin type-I collagen solutions.

**Figure 4:**
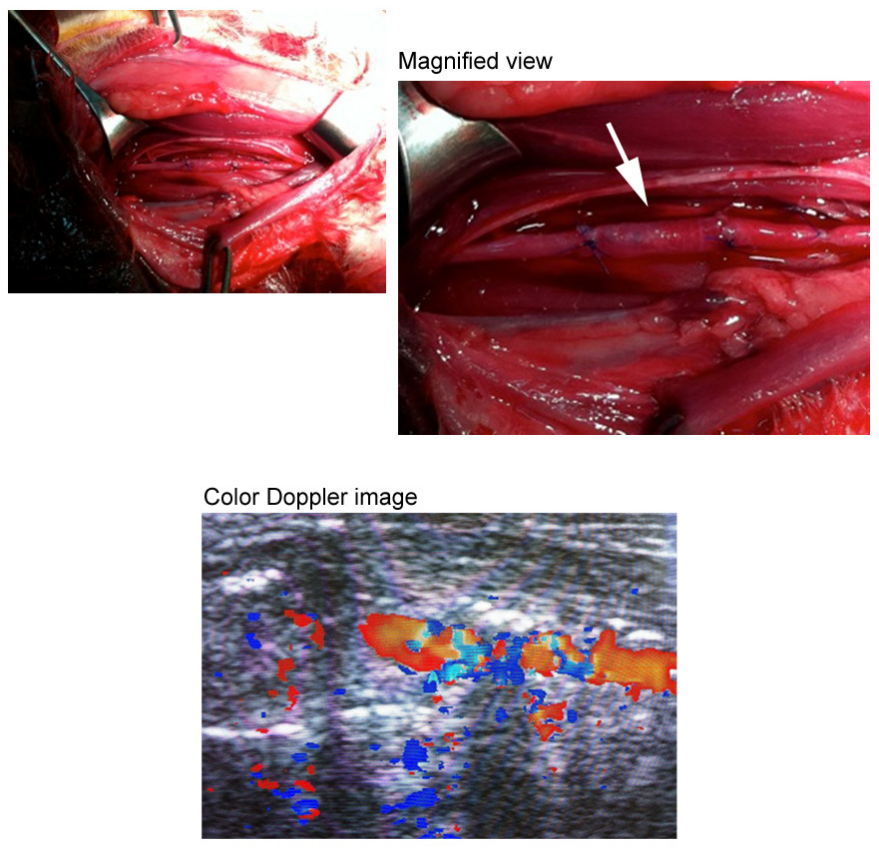
The multi-layered oriented collagen tube transplanted as a blood vessel graft for the carotid artery of a rabbit.

Figure 3 shows photos of various types of three-dimensional oriented collagen scaffolds prepared with rat tail type-I and porcine skin type-I collagen solutions. Figure 3(a) shows typical dehydrated oriented collagen string gels after demineralization. One example of a single-layer dehydrated oriented collagen sheet is shown in Figure 3(b). The direction of orientation of collagen fibrils is the arrayed direction of the string gels. Multi-layered sheets can be prepared by piling up the string gels layer by layer. Neither separation nor dissolution was observed for the connected strings when they were hydrated again even *in vivo* conditions [14]. High density oriented collagen sheets can be produced by arraying half-dried collagen string gels (Figure 3(c)). While the single-layer sheet has 15 strings/cm, the high density one has 50 strings/cm. The density of collagen fibrils remains high when the sheet was hydrated again in PBS. Mesh type oriented collagen sheets can be also prepared by arraying the string gels with a constant interval and dehydrating the gels as shown in Figure 3(d). Due to the orientation of collagen fibrils in the strings, the mechanical strength of the mesh sheet is expected to be higher than that of conventional mesh sheets produced by unoriented collagen fibrils. The mesh sheet can be used by itself and it can be used as an intermediate layer of a multi-layered collagen sheet to improve the strength and elasticity of the sheet. Oriented collagen tubes (Figure 3(e)) can be prepared by rolling up the oriented collagen sheet. The thickness of the tube can be designed by changing the number of rolls and/or using different multi-layered oriented collagen sheets. The minimum inner diameter of the tube can be realized at least down to 0.5 mm using a small diameter rod to be rolled by oriented collagen sheets. It should be noted that the direction of the collagen orientation depends on the direction to which the original oriented collagen sheet will be rolled up. In case of Figure 3(e), the direction of the orientation is the longitudinal direction of the tube. A multi-layered oriented collagen tube was designed using 4 oriented collagen layers as the innermost layer and 6 oriented collagen layers as the outermost layer with 3 mesh sheet layers between them (Figure 3(f)). The intermediate mesh sheet layer can improve the suturability. Figure 4 indicates an example of the suturability of the multi-layered oriented collagen tube which was successfully transplanted as a blood vessel graft for the carotid artery of a rabbit. A color Doppler image of the graft is also shown in Figure 4. Another flexible design of the orientation in a collagen scaffold is shown in Figure 3(g). The dome-shaped sheet has twenty layers inside the scaffold and the orientation of the collagen in each layer is mutually orthogonal. This structure is a simplified simulation of corneal stroma.

Figure 3 also shows photos of oriented collagen scaffolds ((h) strings, (i) single-layer sheet, (j) high density single-layer sheet) prepared with porcine skin type-I collagen solution. There was no major visual difference between the oriented collagen scaffolds prepared by rat tail type-I and by porcine skin type-I.

The simplicity of the designing and producing process above described allows production of oriented collagen scaffolds with arbitrary sizes and arbitrary directions of the orientation, making them applicable to a variety of tissue engineering applications. Even a complex orientation design including a curve orientation can be realized by arraying and piling up the oriented collagen string gels based on the design.

### 3.2. Birefringence Measurements

**Figure 5:**
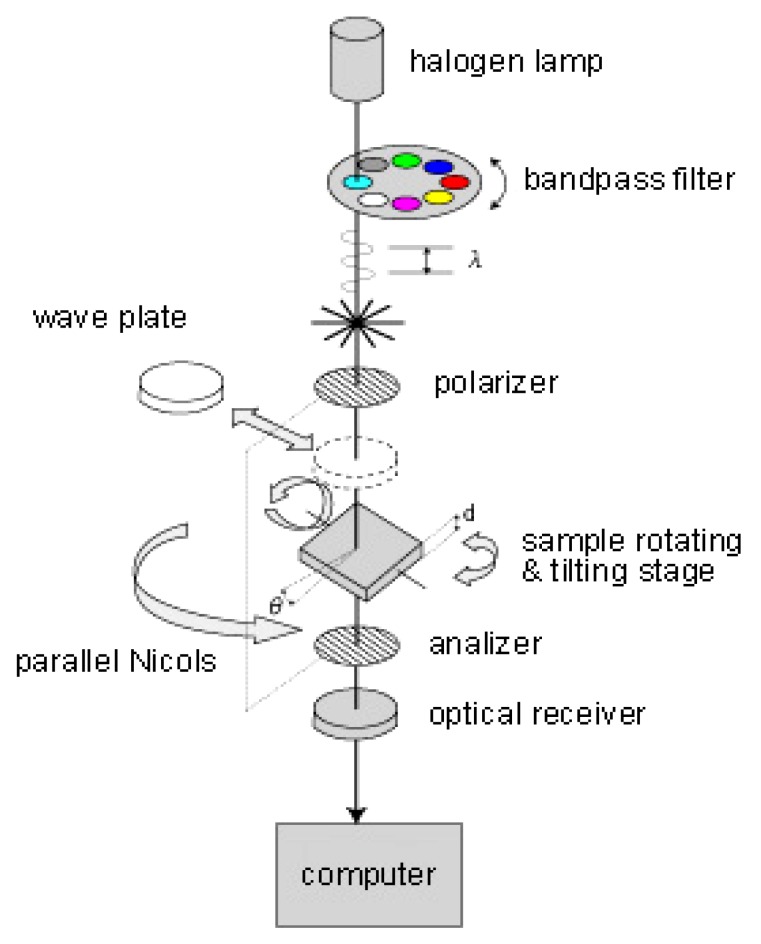
Birefringence measurements by parallel Nicols method.

The structure of collagen can be quantified using optical techniques to express the degree and uniformity of the orientation. Figure 5 illustrates birefringence measurements by parallel Nicols method (KOBRA-CCD, Oji Scientific Instruments, Japan). The oriented collagen scaffolds were viewed between a set of polarizer and analyzer. A bandpass filter was used to obtain a constant wave length of 590 nm. The intensity of light observed through the optical receiver (I(θ)) is expressed by the original intensity of light ($I_{0}$).

(1) $$I\left(\theta \right)=I_{0}(\alpha^{2}\;{cos}^{4}\left(\theta -\varphi \right)+{sin}^{4}\left(\theta -\varphi \right)+\frac{1}{2}C\alpha {sin}^{2}\;2\left(\theta -\varphi \right))$$

(2) $$C=cos\frac{2\pi R}{\lambda }$$

where, θ is rotating angle of a set of polarizer and analyzer, φ is angle of retardation axis of specimen, α is ratio of fraction transmitted over amplitude, R is retardation and λ is wave length.

Birefringence (ΔN) is obtained by the following equation.

(3) ΔN=R/d

where, d is thickness of specimen.

**Figure 6:**
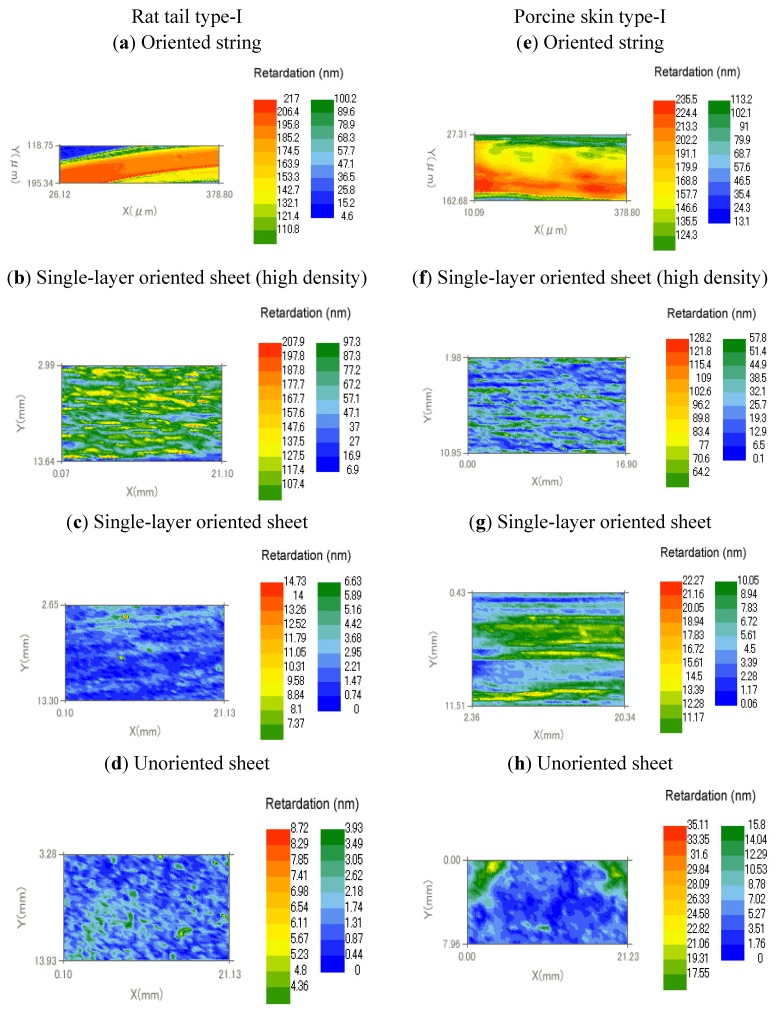
Retardation map of dehydrated oriented collagen scaffolds.

Figure 6 shows retardation map of dehydrated oriented and unoriented collagen scaffolds. The unoriented collagen sheets were prepared by the same process but with a wide nozzle instead of 22 gauge syringe needle. The scaffolds were prepared using two types of collagen solutions, namely, rat tail type-I and porcine skin type-I. Table 1 summarizes the birefringence of collagen scaffolds obtained by average retardations in the map divided by the thickness of the scaffolds. 

**Table 1:** Birefringence of dehydrated oriented collagen scaffolds.

	Type	Collagen	Retardation (nm)	Thickness (μm)	Birefringence (×10^3^)
(a)	Oriented string	Rat tail type-I	195	57	3.4
(b)	Oriented string	Porcine skin type-I	213	92	2.3
(c)	Single-layer oriented sheet (high density)	Rat tail type-I	127	62	2.0
(d)	Single-layer oriented sheet (high density)	Porcine skin type-I	83.4	55	1.5
(e)	Single-layer oriented sheet	Rat tail type-I	3.6	16	0.2
(f)	Single-layer oriented sheet	Porcine skin type-I	13	11	1.2
(g)	Unoriented sheet	Rat tail type-I	1.7	12	0.1
(h)	Unoriented sheet	Porcine skin type-I	5.2	21	0.3

Table 1 indicates that the oriented collagen strings prepared by two collagen solutions of rat tail type-I and porcine skin type-I were highly birefringent. The high density single-layer oriented sheet also revealed high birefringence. The single-layer oriented sheets prepared by the two collagen solutions showed higher birefringence compared with the unoriented collagen sheets.

The birefringence measurements by parallel Nicols method indicated that the developed collagen scaffolds had orientations depending on the preparation process.

## 4. Conclusions

Various types of three-dimensional oriented collagen scaffolds were developed by arraying highly oriented collagen string gels and dehydrating the arrayed gels. To obtain the string gels, concentrated collagen solutions were subjected to shear and extensional flow as they were drawn onto a substrate to induce fibrillogenesis under PBS buffer. Parallel Nicols method was used to confirm the degree of the orientation and to calculate the birefringence of the scaffolds. The process proposed in the present study is flexible to design and produce any size and any direction of orientation for collagen scaffolds used as three-dimensional environments in tissue engineering.

## References

[1] Parenteau-Bareil R., Gauvin R., Berthod F. (2010). Collagen-based biomaterials for tissue engineering applications. Materials.

[2] Ehara A., Ogata K., Imazato S., Ebisu S., Nakano T., Umakoshi Y. (2003). Effects of α-TCP and TetCP on MC3T3-E1 proliferation, differentiation and mineralization. Biomaterials.

[3] Harley B.A., Lynn A.K., Wissner-Gross Z., Bonfield W., Yannas I.V., Gibson L.J. (2010). Design of a multiphase osteochondral scaffold. II. Fabrication of a mineralized collagen-glycosaminoglycan scaffold. J. Biomed. Mater. Res. A.

[4] Cook J.L., Fox D.B., Malaviya P., Tomlinson J.L., Farr J., Kuroki K., Cook C.R. (2006). Evaluation of small intestinal submucosa grafts for meniscal regeneration in a clinically relevant posterior meniscectomy model in dogs. J. Knee Surg..

[5] Yokota T., Ichikawa H., Matsumiya G., Kuratani T., Sakaguchi T., Iwai S., Shirakawa Y., Torikai K., Saito A., Uchimura E., Kawaguchi N., Matsuura N., Sawa Y. (2008). *In situ* tissue regeneration using a novel tissue-engineered, small-caliber vascular graft without cell seeding. J. Thorac. Cardiovasc. Surg..

[6] Lai E.S., Anderson C.M., Fuller G.G. (2011). Designing a tubular matrix of oriented collagen fibrils for tissue engineering. Acta Biomaterialia.

[7] Yost M.J., Baicu C.F., Stonerock C.E., Goodwin R.L., Price R.L., Davis J.M, Evans H., Watson P.D., Gore C.M, Sweet J. (2004). A novel tubular scaffold for cardiovascular tissue engineering. Tissue Engineering.

[8] Zeugolis D.I., Paul R.G., Attenburrow G. (2008). Engineering extruded collagen fibers for biomedical applications. J. Appl. Polym. Sci..

[9] Helary C., Ovtracht L., Coulomb B., Godeau G., Giraud-Guille M. (2006). Dense fibrillar collagen matrices: A model to study myofibroblast behavior during wound healing. Biomaterials.

[10] Berthoda F., Germaina L., Lia H., Xua W., Damourb O., Augera F.A. (2001). Collagen fibril network and elastic system remodeling in a reconstructed skin transplanted on nude mice. Matrix Biology.

[11] Márqueza S.P., Martíneza V.S., Ambrosea W.M., Wanga J., Gantxeguib N.G., Scheinc O., Elisseeffa J. (2009). Decellularization of bovine corneas for tissueengineering applications. Acta Biomaterialia.

[12] Buillesa N., Janin-Manificata H., Malbouyresb M., Justina V., Rovèrea M., Pellegrinic G., Torbetb J., Hulmesb D.J.S., Burillona C., Damoura O. (2010). Use of magnetically oriented orthogonal collagen scaffolds for hemi-corneal reconstruction and regeneration. Biomaterials.

[13] Duncana T.J., Tanakaa Y., Shia D., Kubota A., Quantock A.J, Nishida K. (2010). Flow-manipulated, crosslinked collagen gels for use as corneal equivalents. Biomaterials.

[14] Mikami H., Kuwahara G., Nakamura N., Kondo M., Tanaka M., Yamato M., Kodama S. (2011). Two-Layered Tissue-Engineered Urethra Using Oral Epithelial and Muscle-derived Cells. Presented at the TERMIS-EU 2011 Annual Meeting.

[15] Atala A. (2008). Bioengineered tissues for urogenital repair in children. PediatricResearch.

[16] Rosner B.I., Siegel R.A., Grosberg A., Tranquillo R.T. (2003). Rational design of contact guiding, neurotrophic matrices for peripheral nerve regeneration. Ann. Biomed. Eng..

[17] Albu M.G., Titorencu I., Ghica M.V., Rosario P. (2011). Collagen-based drug delivery systems for tissue engineering. Biomaterials Applications for Nanomedicine.

[18] Riggs C., Vaughan L., Evans G., Lanyon L., Boyde A. (1993). Mechanical implications of collagen fibre orientation in cortical bone of the equine radius. Anat Embryol.

[19] Jones S.J., Boyde A., Pawley J.B. (1975). Osteoblasts and collagen orientation. Cell Tiss. Res..

[20] Popescu D., Lems R., Rossi N., Yeoh C., Loos J., Holder S., Bouten C., Sommerdijk N. (2005). The patterning and alignment of muscles cells using the selective adhesion of poly(oligoethylene glycol methyl ether methacraylate)-based ABA block copolymers. Adv. Mater..

[21] Thompson D.M., Buettner H.M. (2006). Neurite outgrowth is directed by Schwann cell alignment in the absence of other guidance cues. Ann. Biomed. Eng..

[22] Thompson D.M., Buettner H.M. (2004). Oriented Schwann cell monolayers for directed neurite outgrowth. Ann. Biomed. Eng..

[23] Wilson D.L., Martin R., Hong S., Cronin-Golomb M., Mirkin C.A., Kaplan D.L. (2001). Surface organization and nanopatterning of collagen by dip-pen lithography. Proc. Natl. Acad. Sci. USA.

[24] Knight D.P., Nash L., Hu X.W., Haffegee H., Ho M.-W.J. (1998). *In vitro* formation by reverse dialysis of collagen gels containing highly oriented arrays of fibrils. Biomed. Mater. Res..

[25] Torbet J., Malbouyres M., Builles N., Justin V., Roulet M., Damour O., Oldberg A., Ruggiero F., Hulmes D. (2007). Orthogonal scaffold of magnetically aligned collagen lamellae for corneal stroma reconstruction. J. S. Biomaterials..

[26] Dickenson R.B., Guido S., Tranquillo R.T. (1994). Biased cell migration of fibroblasts exhibiting contact guidance in oriented collagen gels. Ann. Biomed. Eng..

[27] Guo C., Kaufman L. (2007). Flow and magnetic field induced collagen alignment. J. Biomaterials.

[28] Zhong S., Teo W.E., Zhu X., Beuerman R.W., Ramakrishna S., Yung L.Y.L. (2006). An aligned nanofibrous collagen scaffold by electrospinning and its effects on *in vitro* fibroblast culture. J. Biomed. Mater. Res. A.

[29] Kirkwood J.E., Fuller G.G. (2009). Liquid Crystalline Collagen: A self-assembled morphology for the orientation of mammalian cells. Langmuir.

